# Supplementary material for: AKT1 Is Required for a Complete Palbociclib-Induced Senescence Phenotype in BRAF-V600E-Driven Human Melanoma
Source: Cancers (Basel). 2022 Jan 23;14(3):572. doi: 10.3390/cancers14030572 (PMC8833398; doi:10.3390/cancers14030572)
Supplement: Supplementary file 1 [file cancers-14-00572-s001.zip › cancers-1563547-suppl.pdf]

cancers

Supplementary Materials

AKT1 Is Required for a Complete Palbociclib-Induced Senescence Phenotype in BRAF-V600E-Driven Human Melanoma

Abraham L. Bayer, Jodie Pietruska, Jaymes Farrell, Siobhan McRee, Pilar Alcaide and Philip W. Hinds

Table S1. gRNA and primer sequences.

Target (gRNA)	Forward	Reverse
NT	CACCGGCGATCGGAGTGCCACGATA	AAACTATCGTGGCACTCCGATCGCC
AKT1	CACCGTGTGCCGCAAAAGGTCTTCA	AAACTGAAGACCTTTTGCGGCACAC
AKT2	CACCGGCCAGCTGATGAAGACCGAG	AAACCTCGGTCTTCATCAGCTGGCC
AKT3	CACCGTAAGGTAAATCCACATCTTG	AAACCAAGATGTGGATTTACCTTAC
**Target (qPCR)**	**Forward**	**Reverse**
GAPDH	ATGGTGAAGGTCGGTGTGAACG	TGGTGAAGACGGCAGTAGACTC
Il1β	ATGATGGCTTATTACAGTGGCAA	GTCGGAGATTCGTAGCTGGA
Il6	AACCTGAACCTTCCAAAGATGG	TCTGGCTTGTTCCTCACTACT
Il8	ACTGAGAGTGATTGAGAGTGGAC	AACCCTCTGCACCCAGTTTTC
cGAS	TAACCCTGGCTTTGGAATCAAAA	TGGGTACAAGGTAAAATGGCTTT
STING	AGCATTACAACAACCTGCTACG	GTTGGGGTCAGCCATACTCAG
IFNα	GCCTCGCCCTTTGCTTTACT	CTGTGGGTCTCAGGGAGATCA
IFNβ	ATGACCAACAAGTGTCTCCTCC	GGAATCCAAGCAAGTTGTAGCTC

A

Cell Line	InDel	% of Sequences	Resulting Mutation
AKT1 KO Pool	0	10	None
	+1	65	Frameshift
	-1	5	Frameshift
	-3	10	Loss of M306 or K307
	-9	7	Loss of M306, K307, T308
AKT1 Clone D11	+1	97	Frameshift
AKT1 Clone E11	+1	98	Frameshift

B

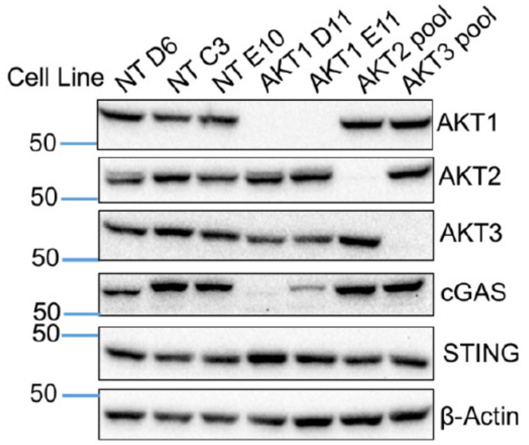

Figure S1. Characterization of AKT1 Clones. **A**. Table of mutations found by TIDE analysis of pooled AKT1 gRNA treated cells and AKT1 clones. **B**. Western blot showing isoform specific knock-out and cGAS/STING levels in all clonal cell lines, and AKT2/AKT3 pooled cells.

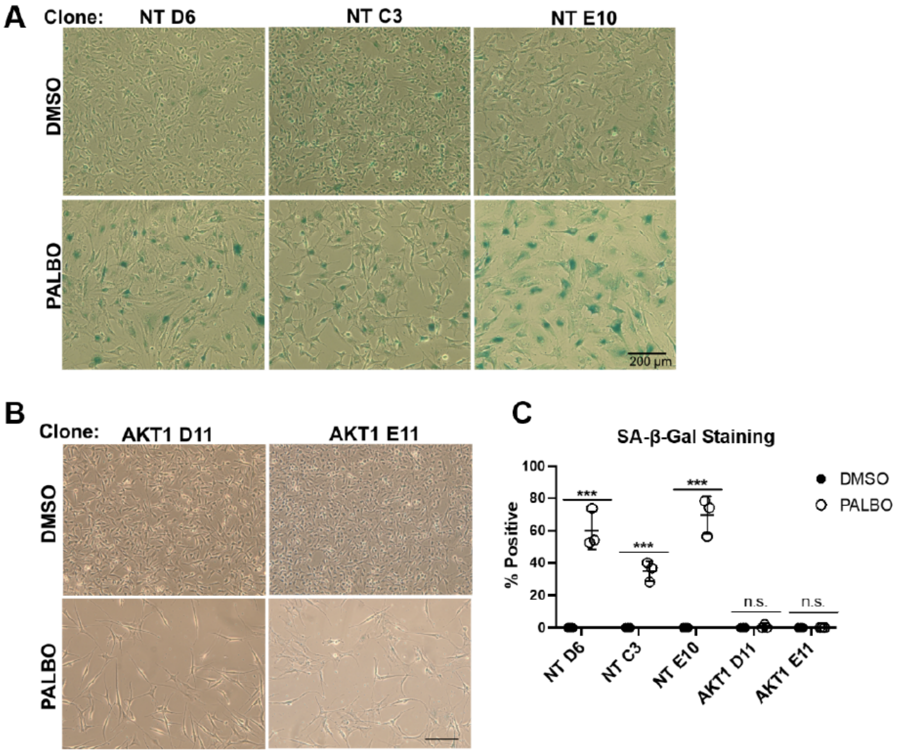

Figure S2. NT and AKT1 Clones Display Similar Senescence Phenotypes. Beta-galactosidase staining after 7 days treatment with PALBO at 1 µM or DMSO as a control in 3 independent NT clones (**A**) and 2 independent AKT1 clones (**B**), quantified in (**C**).

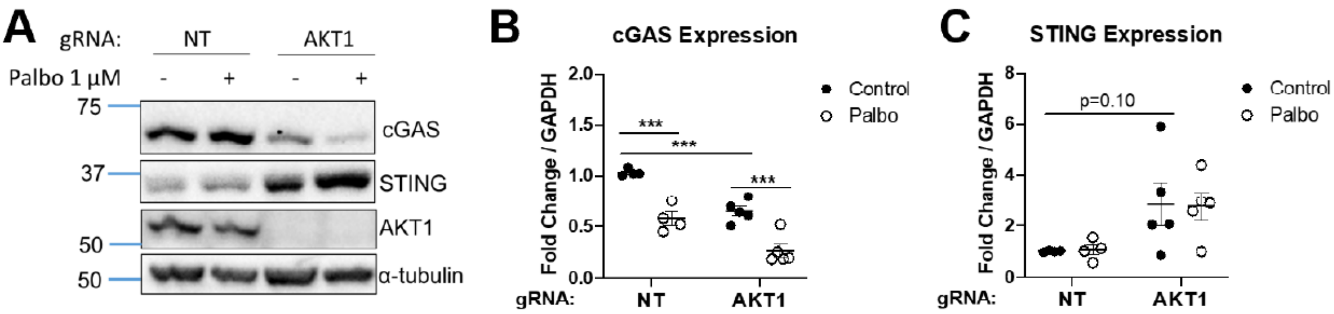

Figure S3. Effect of Palbociclib on cGAS & STING. **A**. Representative western blot for cGAS and STING of cells treated with PALBO or DMSO for 1 week at 1.0 µM. Expression of cGAS (**B**) & STING (**C**) by RT-qPCR in PALBO or DMSO treated cells. Significance determined by Student's *T*-test (* = $p < 0.05$, ** = $p < 0.01$, *** = $p < 0.001$, ns = no significance).

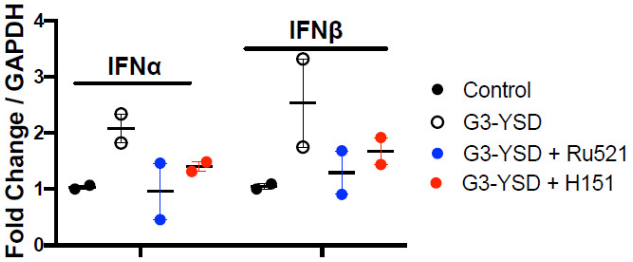

Figure S4. Ru521 + H151 are Functional. A. IFNα/β induction by RT-qPCR in NT WM1799 cells transfected with G3-YSD for 48 hours, with or without a 2 hour pre-treatment with Ru521 (2.5 µM) or H151 (5.0 µM).

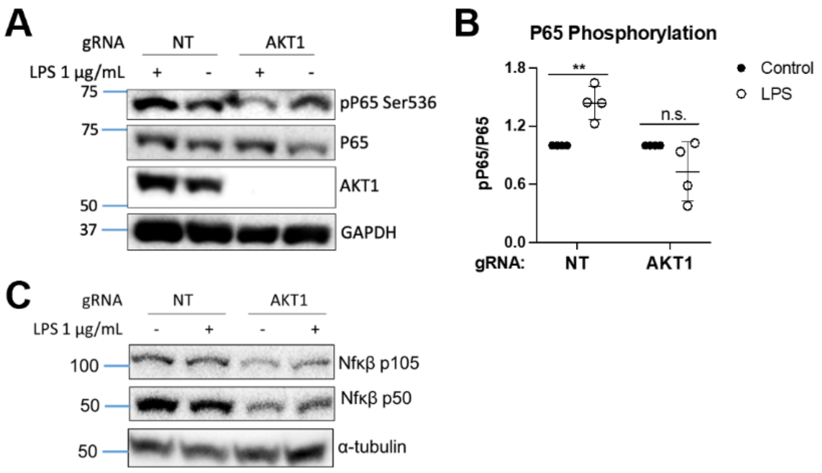

Figure S5. AKT1 Knockout Impairs NF-kB Signaling. **A**–**C**. Representative western blots of cells treated with LPS for 90 minutes at 1 μg/mL with quantification of phospho-P65 over total P65 normalized to untreated cells for n=4 independent experiments of p65 phosphorylation. Significance determined by Student's T-test (* = $p < 0.05$, ** = $p < 0.01$, *** = $p < 0.001$, ns = no significance).
