# Supplementary material for: AKT1 Is Required for a Complete Palbociclib-Induced Senescence Phenotype in BRAF-V600E-Driven Human Melanoma
Source: Cancers (Basel). 2022 Jan 23;14(3):572. doi: 10.3390/cancers14030572 (PMC8833398; doi:10.3390/cancers14030572)
Supplement: Supplementary file 1 [file cancers-14-00572-s001.zip › Uncropped WBs.pdf]

AKT1 is Required for a Complete Palbociclib-Induced Senescence Phenotype in BRAF-V600E-Driven Human Melanoma

Supplementary Material: Uncropped Western Blots

Figure 1A:

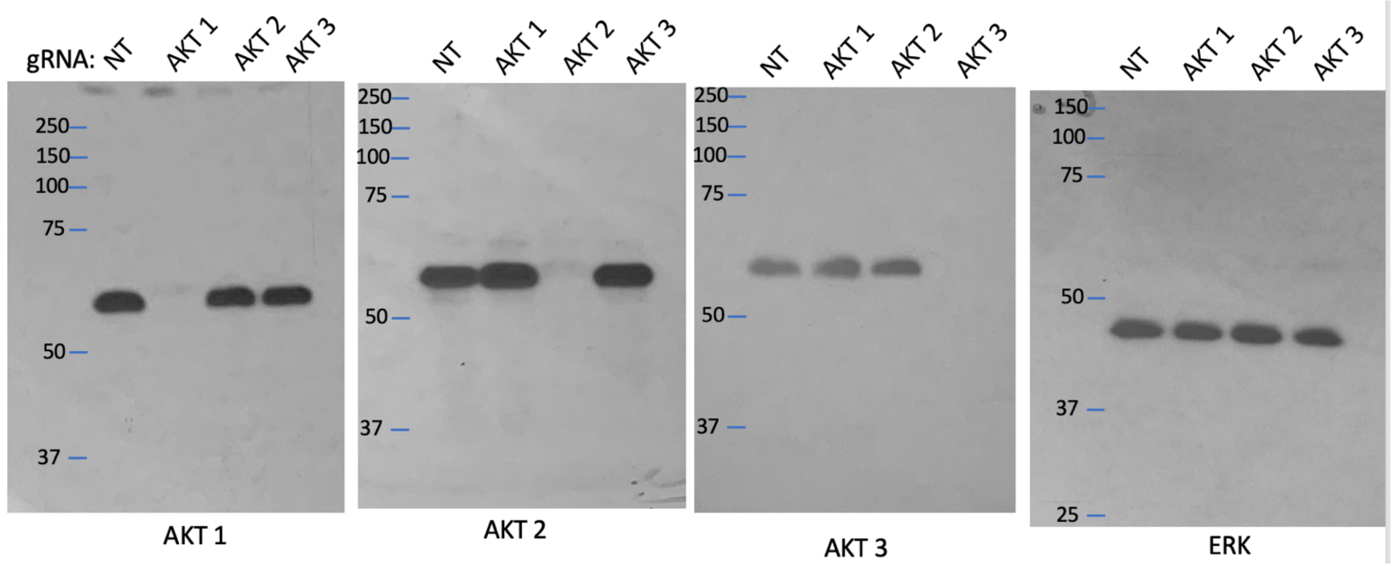

Figure 2F:

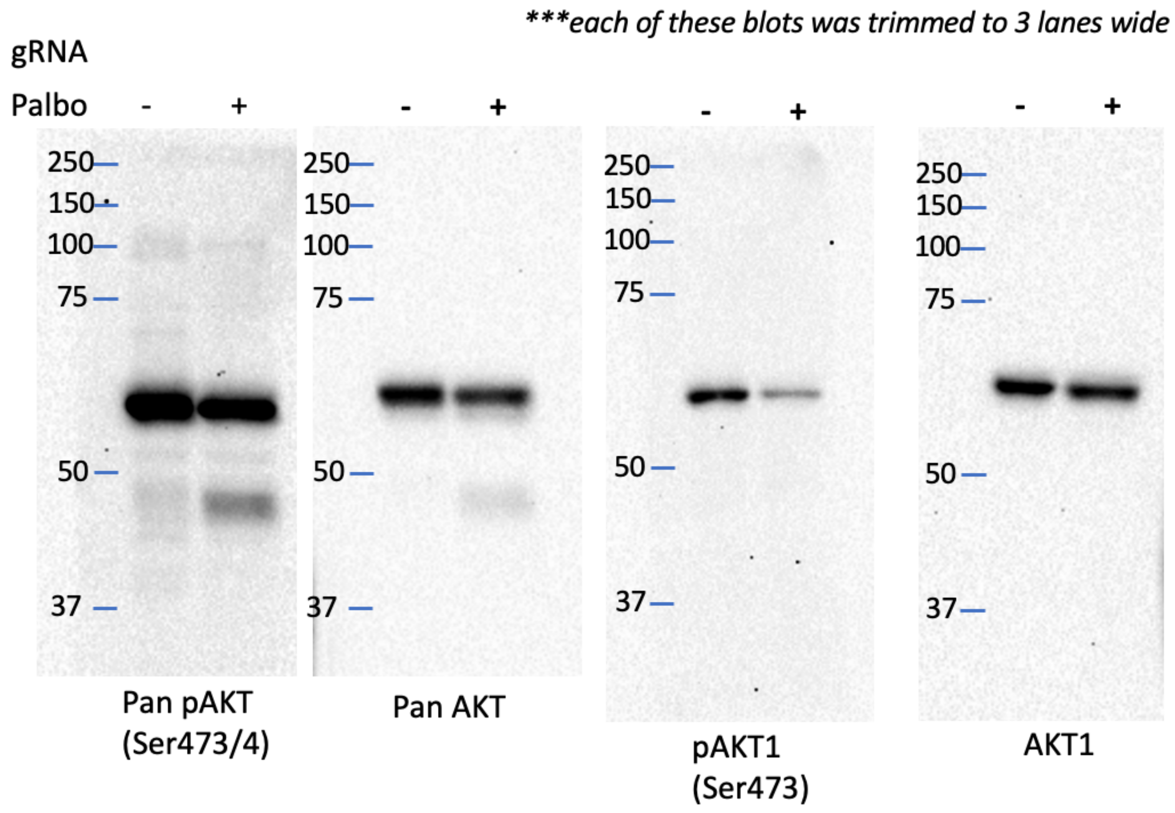

Figure 2F cont.

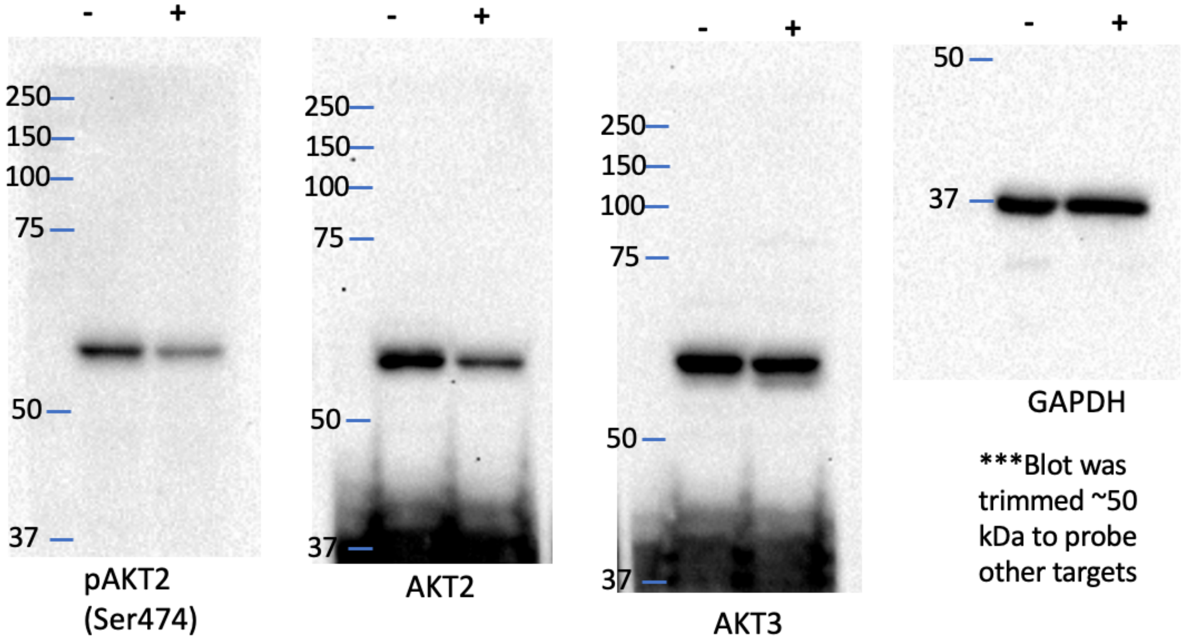

Figure 2I:

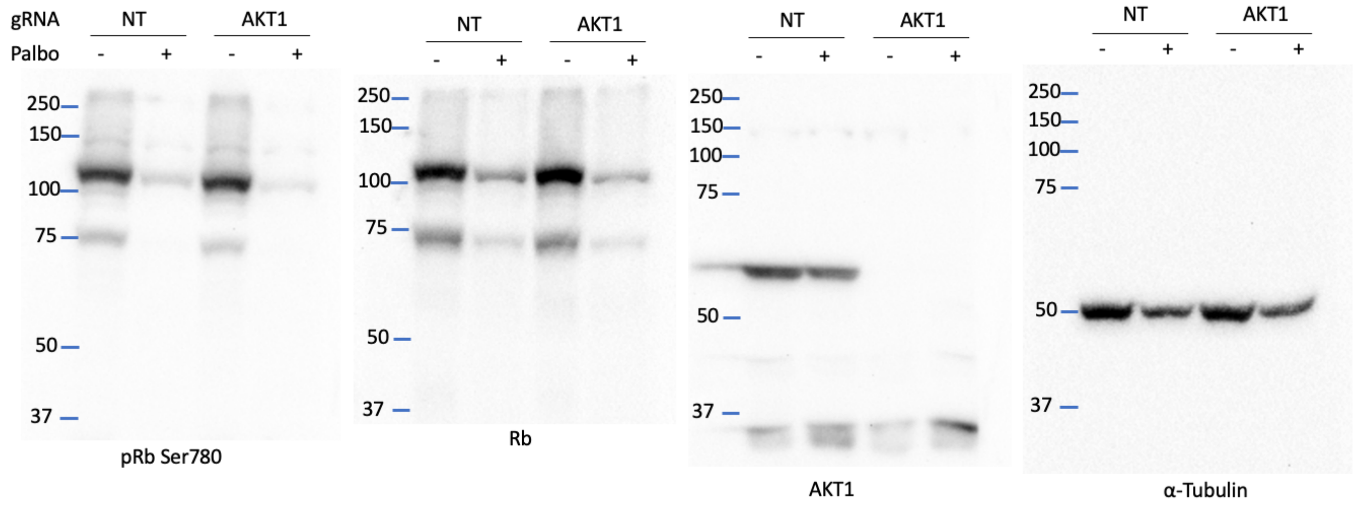

Figure 3A:

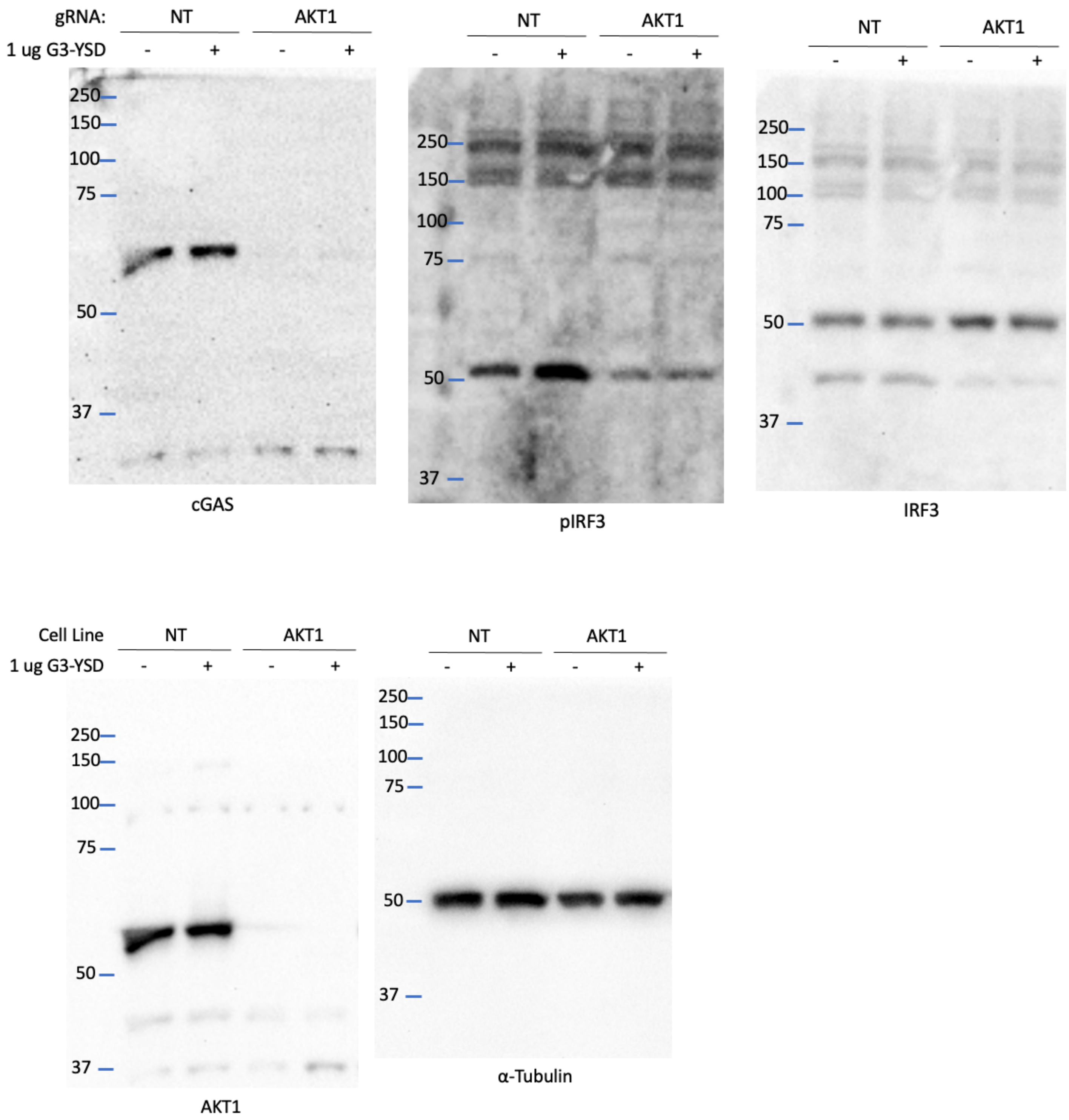
gRNA:
NT
AKT1
1 ug G3-YSD
- + - +
250
150
100
75
50
37
cGAS
pIRF3
IRF3
Cell Line
AKT1
α-Tubulin

Figure 4A:

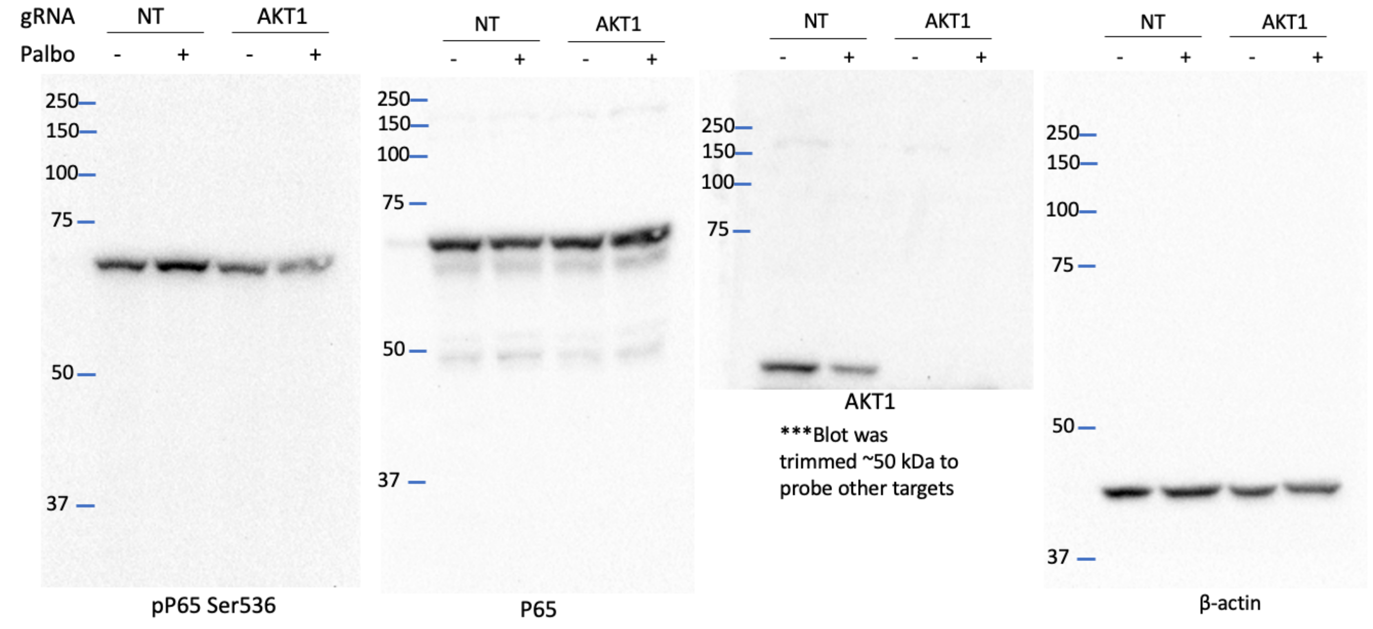

Figure 4B:

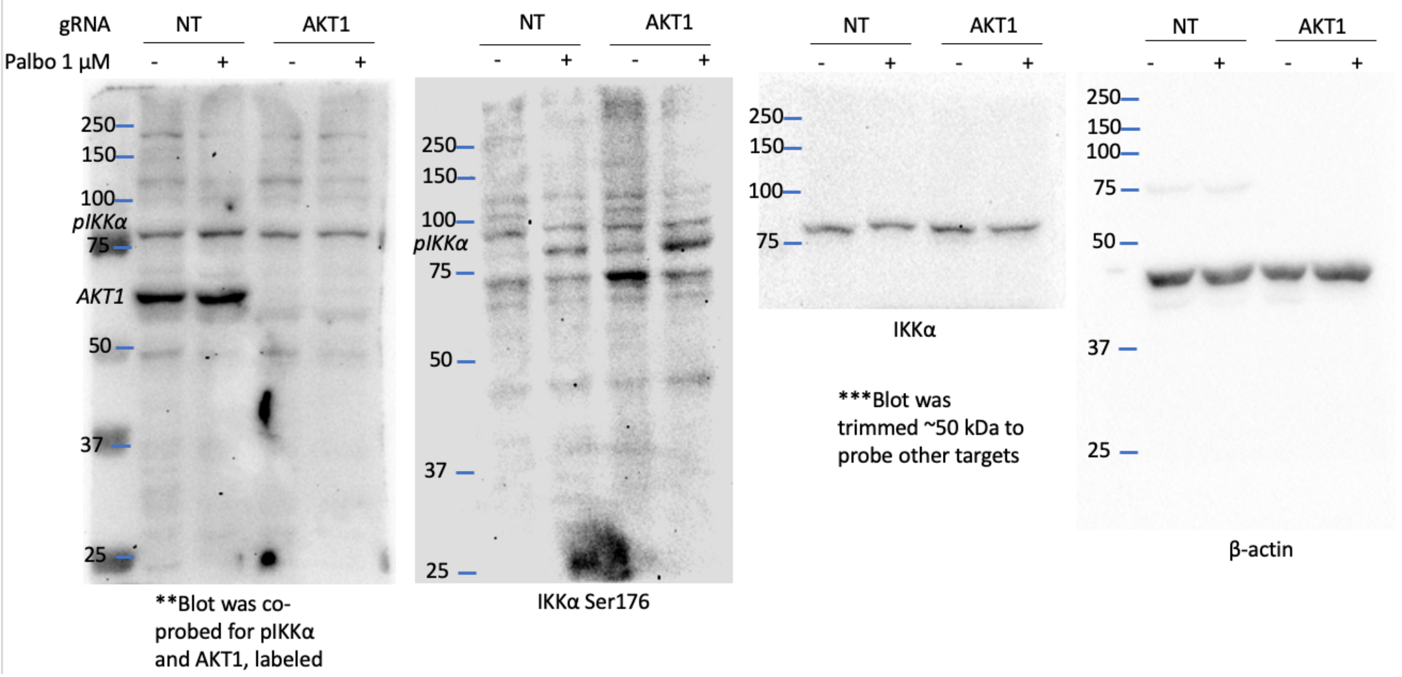

Figure 4H:

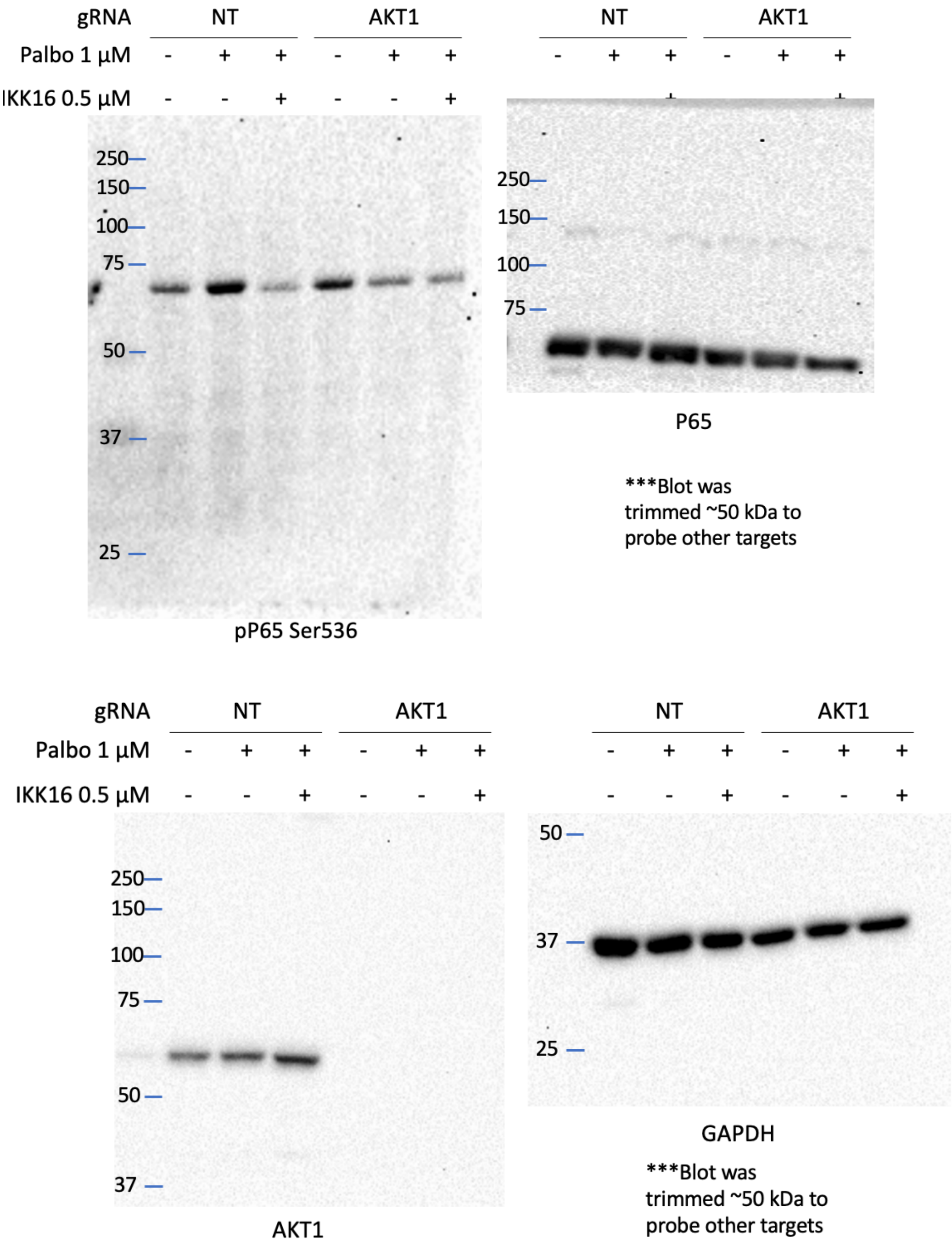
gRNA
NT
AKT1
NT
AKT1
Palbo 1 µM
- + + - + +
- + + - + +
IKK16 0.5 µM
- - + - - +
250
150
100
75
50
37
25
pP65 Ser536
250
150
100
75
P65
***Blot was trimmed ~50 kDa to probe other targets
gRNA
NT
AKT1
NT
AKT1
Palbo 1 µM
- + + - + +
- + + - + +
IKK16 0.5 µM
- - + - - +
- - + - - +
250
150
100
75
50
37
AKT1
50
37
25
GAPDH
***Blot was trimmed ~50 kDa to probe other targets

Figure 4I:

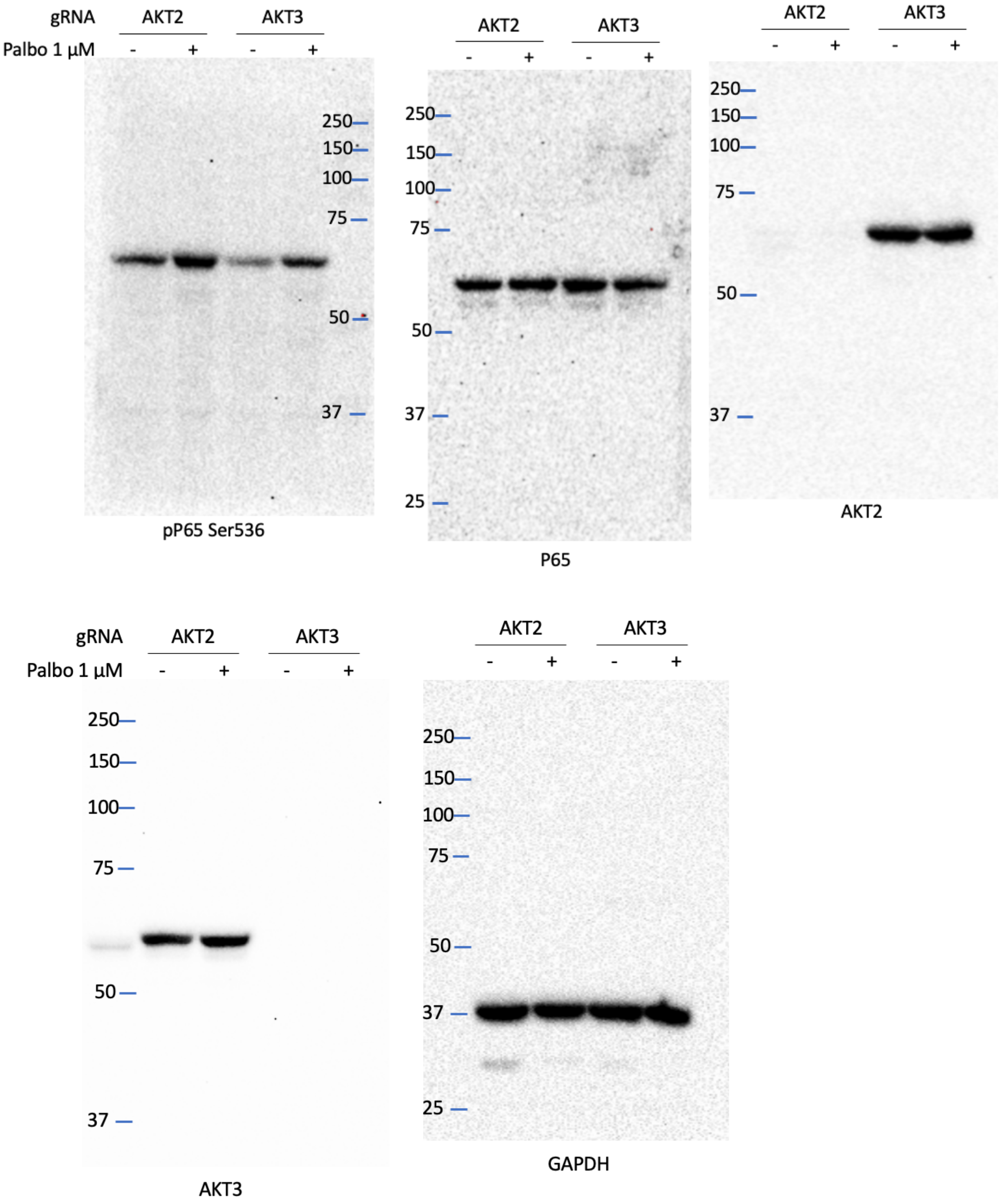
gRNA
AKT2
AKT3
Palbo 1 µM
- + - +
250
150
100
75
50
37
pP65 Ser536
AKT2
AKT3
- + - +
250
150
100
75
50
37
25
P65
AKT2
AKT3
- + - +
250
150
100
75
50
37
AKT2
gRNA
AKT2
AKT3
Palbo 1 µM
- + - +
250
150
100
75
50
37
AKT3
AKT2
AKT3
- + - +
250
150
100
75
50
37
25
GAPDH

Figure S1B:

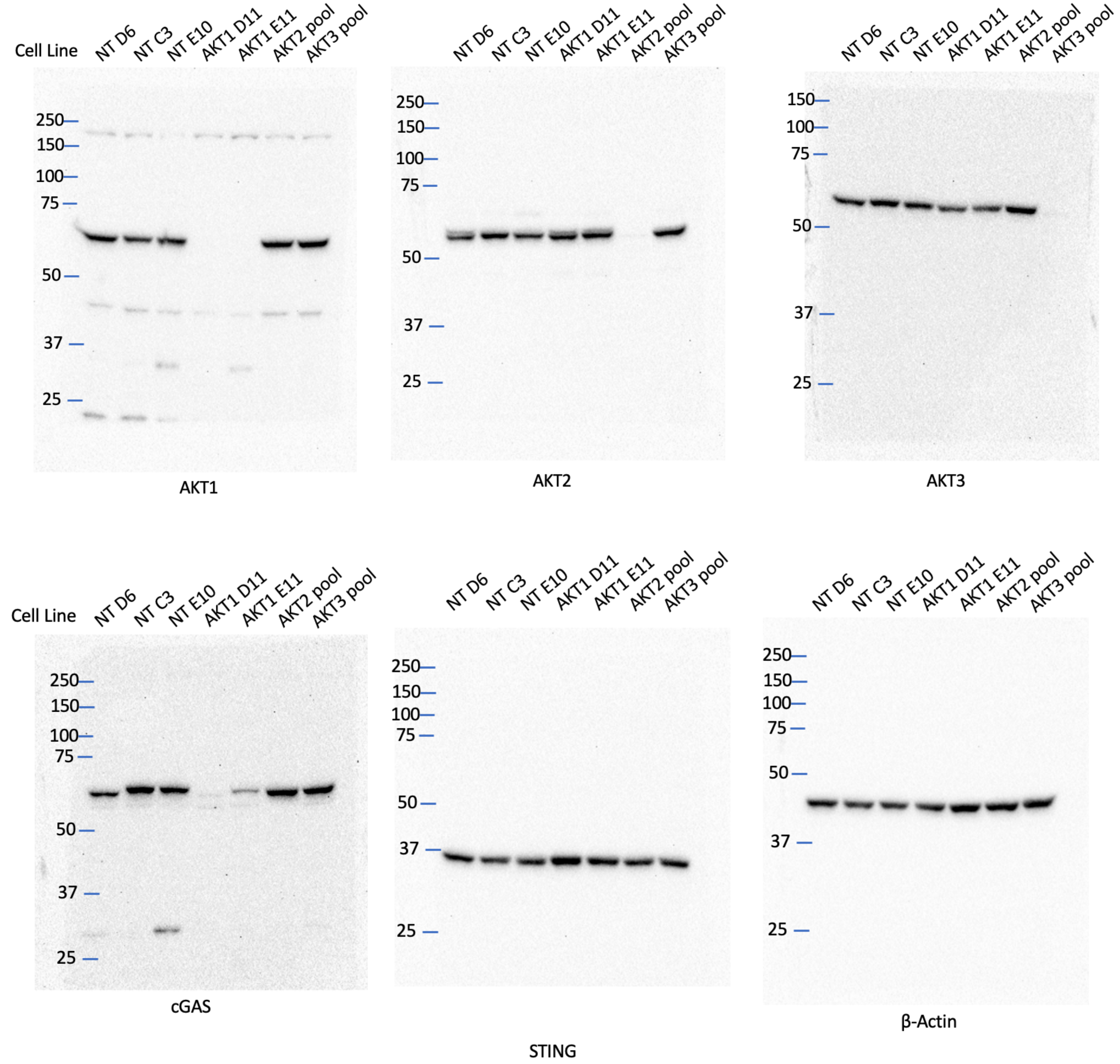
Cell Line
NT D6
NT C3
NT E10
AKT1 D11
AKT1 E11
AKT2 pool
AKT3 pool
250
150
100
75
50
37
25
AKT1
AKT2
AKT3
cGAS
STING
β-Actin

Figure S3A:

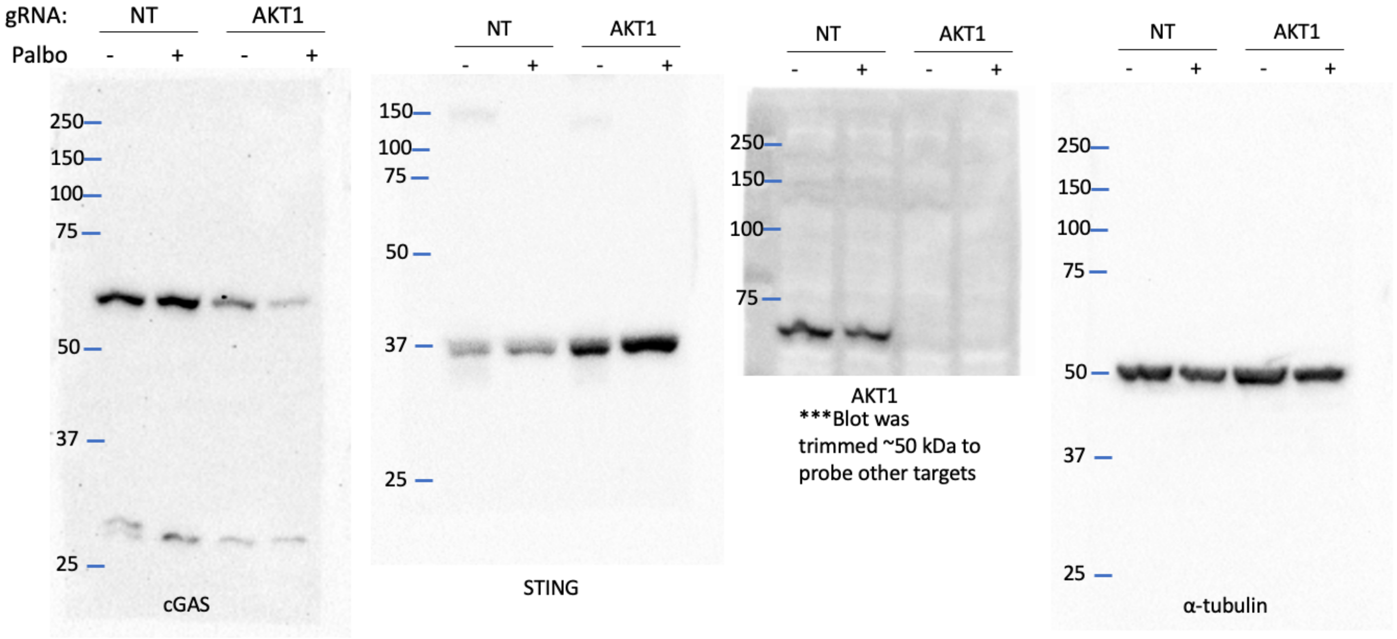

Figure S5A:

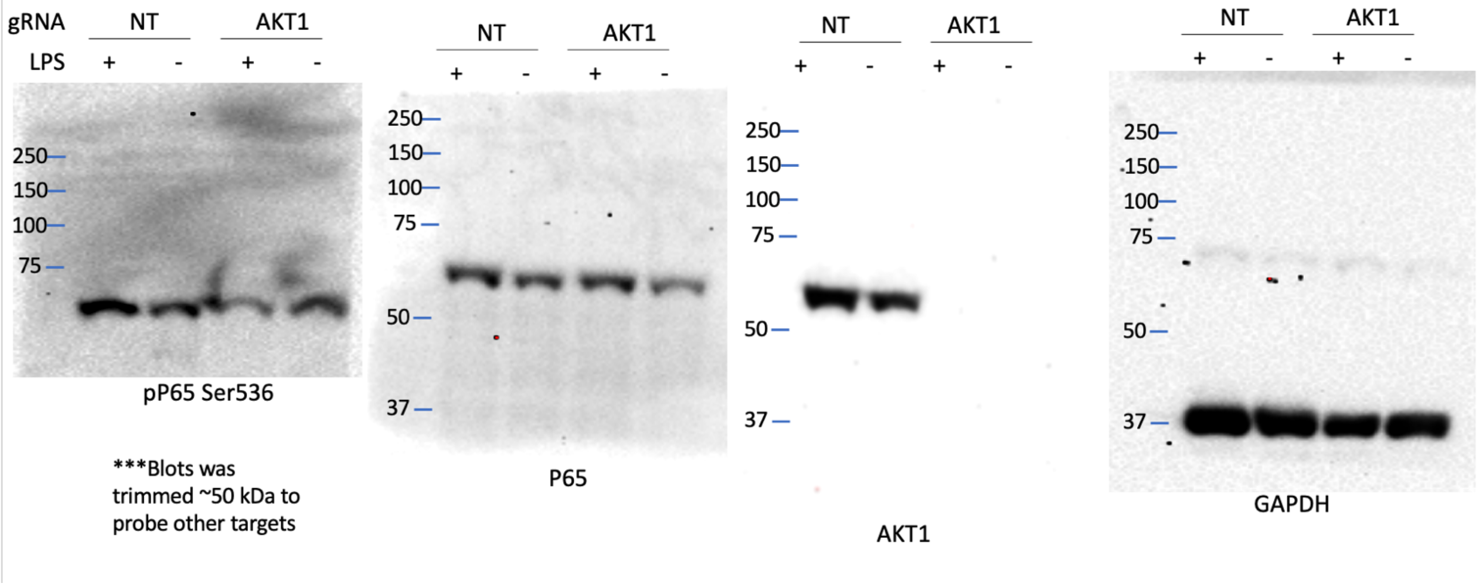

Figure S5C:

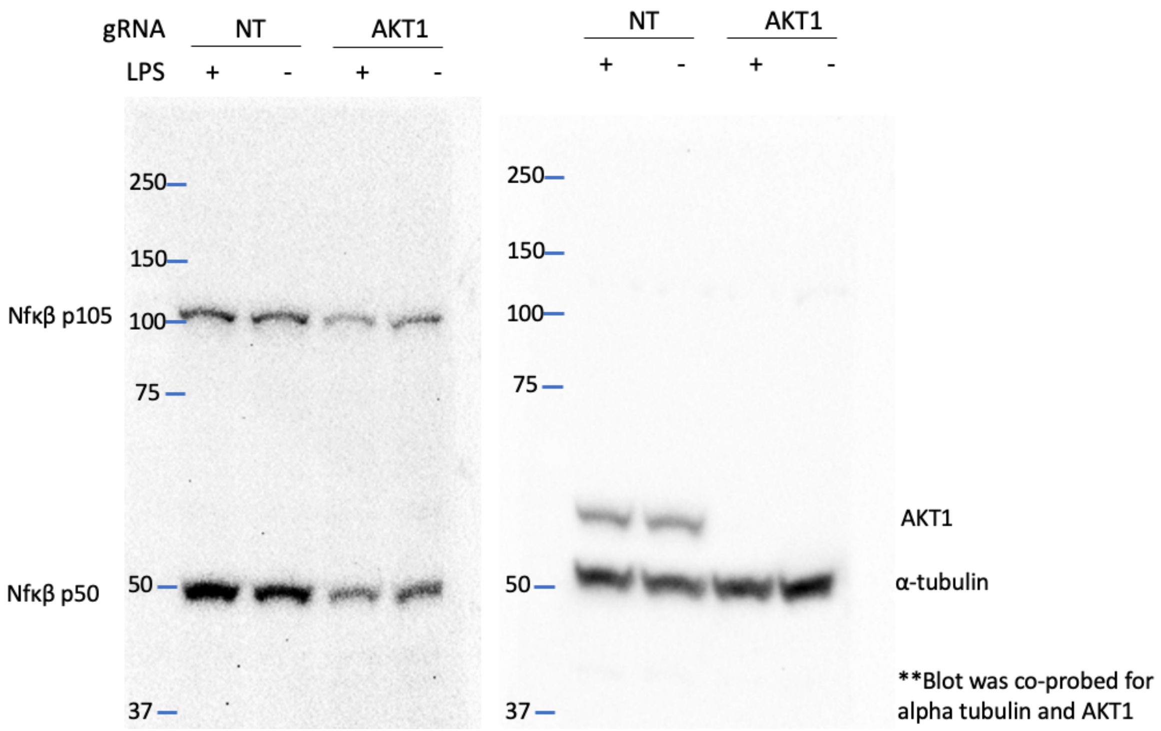
gRNA
NT
AKT1
LPS
+
-
+
-
250
150
100
75
50
37
Nfκβ p105
Nfκβ p50
NT
AKT1
+
-
+
-
250
150
100
75
50
37
AKT1
α-tubulin
**Blot was co-probed for
alpha tubulin and AKT1
